# The complexity of the interaction between binge-eating and attention

**DOI:** 10.1371/journal.pone.0215506

**Published:** 2019-04-24

**Authors:** Roni Halevy-Yosef, Eytan Bachar, Lilach Shalev, Yehuda Pollak, Adi Enoch-Levy, Eitan Gur, Abraham Weizman, Daniel Stein

**Affiliations:** 1 Department of Psychology, The Hebrew University of Jerusalem, Jerusalem, Israel; 2 Safra Children's Hospital, Sheba Medical Center, Tel Hashomer, Israel; 3 Department of Psychiatry, Hadassah Medical Center, Jerusalem, Israel; 4 School of Education and School of Neuroscience, Tel-Aviv University, Tel Aviv, Israel; 5 The Seymour Fox School of Education, The Hebrew University of Jerusalem, Jerusalem, Israel; 6 Sheba Medical Center, Tel Hashomer, Israel; 7 Geha Mental Health Center and Felsenstein Medical Research Center, Rabin Medical Center, Petah Tiqva, Israel; 8 Department of Psychiatry, Sackler Faculty of Medicine, Tel Aviv University, Tel Aviv, Israel; QIMR Berghofer Medical Research Institute, AUSTRALIA

## Abstract

**Objective:**

To investigate whether binge-eating in patients with eating disorders (EDs) is associated with attentional deficits.

**Methods:**

We studied ED patients with binge-eating (n = 51), no binge-eating (n = 59) and controls (n = 58). ED patients were assessed following the stabilization of weight and ED pathology. Attention assessment included evaluation of attention deficit hyperactivity disorder (ADHD) diagnosis, the Adult ADHD Self-Report (ASRS) and ADHD Rating Scale-IV-Home Version (ADHD-RS) questionnaires, and attention functioning assessed with neuropsychological tools. The severity of eating-related pathology, depression, anxiety and obsessionality was also monitored.

**Results:**

Patients with binge-eating showed more ADHD symptomatology on the ADHD-RS compared with non-binge-eating patients. No differences were found between binge-eating and non-binge-eating patients in ADHD diagnosis and neuropsychological functioning. Among the specific ED subtypes, patients with anorexia nervosa binge/purge type (AN-B/P) showed the highest rates of ADHD symptomatology on the ADHD-RS, and were characterized with sustained attention deficits.

**Conclusion:**

Binge-eating is not associated with attention deficits as measured by objective neuropsychological tools. Nonetheless, it is associated with attentional difficulties as measured with the self-reported ADHD-RS. AN-B/P patients are the only ED category showing objective sustained attention deficits.

## Introduction

The association between eating disorders (EDs) and attention deficit hyperactivity disorder (ADHD) is a subject of great interest in recent years [1,2]. Epidemiological studies show elevated rates of ADHD in ED patients, specifically in patients with binge-eating [3,4,5,6]. Second, elevated rates of disordered eating and EDs, primarily bulimia nervosa (BN), have been found in patients with ADHD in comparison with normal controls [7,8,9]. Third, case reports of patients with comorbid BN and ADHD show beneficial effects of psychostimulants in reducing the rate and severity of binge/purge behaviors and in increasing personal control over eating [10,11].

The notion that cognitive impairment such as attention deficit plays a role in the development of bingeing behavior [6,12] may have practical implications in the treatment of both binge-eating [13] and ADHD in patients with EDs [14]. Nonetheless, it is important to understand whether patients with binge-type EDs are characterized by objective attention deficits. Alternatively, attention difficulties may be secondary to impulsivity, emotional dysregulation, lack of self-awareness and risk-taking behaviors such as substance abuse, that are all characteristic of patients with BN [12,13,15].

To better understand this issue, we explored the association of attentional deficits and binge-eating in patients with BN, as this is an ED category with binge-eating and normal weight range (see DSM- (5[16]. Reviewing the literature, we found only a few neuropsychological studies assessing attention functions in patients with BN. Some studies [17,18,19], although not all [20,21,22], suggested that attention deficits might exist in patients with BN. Nonetheless, these studies explored only one or two functions of attention (mainly sustained attention) and suffered from methodological inconsistencies, mainly the use of different assessment batteries [17,18,19].

Considering these inconsistent results, we sought to explore whether neurocognitive attentional deficits would be associated with binge-eating, going beyond the mere artifacts of the shared symptomatology of ADHD and binge/purge type EDs. For this purpose, we utilized an ADHD theoretical framework defining attention as a complex system, focusing on four attentional functions [23,24]: (a) sustained attention, i.e., the ability to allocate attentional resources to a non-attractive task over time while maintaining a constant level of performance; (b) selective-spatial attention, i.e., the ability to focus attention on a relevant target while ignoring adjacent distracters; (c) orienting of attention, i.e., the ability to direct attention over the visual or auditory field according to sensory input, and to disengage and reorient efficiently; and (d) executive attention, i.e., the ability to resolve conflicts of information and/or responses. For a comprehensive assessment of ADHD, we also used a clinical interview to estimate the prevalence of ADHD, and self-report questionnaires to assess the severity of ADHD symptoms. The severity of comorbid symptoms (depression, anxiety and obsessionality), potentially affecting neuropsychological performance, was also monitored.

At the start, we hypothesized that patients with any type of ED will report greater frequency and/or severity on the self-rating ADHD scales in comparison to control participants. Second, we hypothesized that patients with EDs involving binge-eating will report higher frequency of ADHD diagnosis and more severe self-rated ADHD symptoms and will show more impaired performance on the attention tests compared to patients with non-binge type EDs and controls.

## Methods

### Participants

One-hundred-sixty-eight female adolescents and young adults participated in the study: 51 with DSM-5 [16] EDs involving binge-eating (33 with BN and 18 with anorexia nervosa binge/purge type [AN-B/P]), 59 with DSM-5 [16] EDs not involving binge-eating (34 with AN-restricting type [AN-R], 18 with AN-purging type, and 7 with purging disorder [PD]), and 58 control subjects.

The following were our inclusion criteria: female gender; age: 15–28 years; good understanding of the Hebrew language; normal visual acuity and no motor impairment. Exclusion criteria were lifetime or current schizophrenic spectrum disorders, bipolar disorders, substance use disorders, organic–brain disorders, mental retardation, and lifetime or current medical illnesses potentially affecting appetite or weight. All patients were hospitalized at the time of the evaluation in the adolescent and adult ED inpatient departments of the Sheba Medical Center, Tel Hashomer, Israel.

Control participants included age-matched healthy volunteer females, who had no lifetime or current history of any psychiatric or medical disorder and no regular use of medications. Their lifetime and current weight was above 90% of average body weight, based on the 2000 sex-specific growth charts from the CDC (www.cdc.gov/growthcharts) for adolescents, found adequate for Israeli youngsters [25], and the Metropolitan Life Insurance chart (1983) for adults [26]. All control females had regular menses since menarche. Adolescent control participants were recruited by snowball sampling method. Adult controls were recruited by advertisements distributed in several universities in Israel.

### Instruments

Diagnosis of EDs and comorbid psychiatric disorders was determined according to the DSM-5 [16] criteria, using the Structured Clinical Interview for DSM-IV Axis I Disorders-Patient Edition (SCID-I/P Version 2.0) [27]_,_ adapted for DSM-5 [16]. Comorbidity of ADHD was assessed using the ADHD module of the schedule for affective disorders and schizophrenia for school- age children-present and lifetime version (K-SADS-PL) [28], previously used also in adolescent and adult Israeli populations [29].

Control participants have been interviewed using the 10 general screening criteria of the SCID-I/P Version 2.0 [27], as well as the SCID-I/P Version 2.0 screening items for depressive disorders, bipolar disorders, and schizophrenic spectrum disorders. Each screening item of the SCID-I/P Version 2.0 is rated as either present, questionable and not present. Only control participants answering negatively on all SCID- I/P Version 2.0 screening items have been included the study. This tool has been previously used in differentiating controls from patients with EDs [30]. Controls have been further screened for ED-related symptoms with the SCOFF, [31] previously used in Israeli populations [32]. In the present design, we have excluded control participants answering positively on any one item of the SCOFF. Last, controls have been screened for ADHD symptoms using the screening items of the ADHD module of the K-SADS [28]. Only control participants answering negatively on all screening items have been included the study.

#### Demographic and clinical data

Age, years of education and countries of birth were assessed using a structured questionnaire. Data about duration of illness, duration of inpatient treatment, weight, height, body mass index (BMI), and medications administered during inpatient treatment was collected from the patients' medical records. Medications used were divided into antidepressants, antipsychotics, mood stabilizers, and psychostimulants.

#### Assessment of psychopathology

**Questionnaires.** The *Eating Disorders Examination-Questionnaire* version 6.0 (EDE-Q) [33,34] includes 36 items converging to four scales assessing different restricting behaviors (restraint, eating concerns, shape concerns and weight concerns), and 11 items assessing bingeing/purge behaviors. The internal consistency of the different EDE-Q scales in the present study ranged from 0.87 to 0.97.

*The 26-item Eating Attitudes Test-26* (EAT-26) [35] includes 26 items assessed core eating-related pathology. The Total EAT-26 score was used as a measure of ED severity. The internal consistency of the EAT-26 in the present study was 0.96.

We also used two scales of the 91-items *Eating Disorder Inventory-*2 (EDI-2) [36] considered relevant for our purposes: the EDI-2-Interoceptive Awareness (EDI-2-IA), assessing the ability to recognize, identify and express internal emotional and perceptual states, and the EDI-2-Impulse Regulation (EDI-2-IR), assessing ED-related impulsivity. The internal consistency of the EDI-2-IA and EDI-2-IR in the present study was 0.89.

Depression was assessed using the 21-items *Beck Depression Inventory* (BDI) [37]; anxiety was assessed using the 40-items *State-Trait Anxiety Inventory* (STAI) [38], measuring the severity of anxiety at the time of examination (STAI-State), and the general tendency to display anxiety (STAI-Trait). In the present study, we related only to findings on the STAI-Trait. The internal consistency of the BDI and the STAI-Trait in the present study was 0.95 and 0.96, respectively.

Severity of obsessionality was assessed using the 20-items *Leyton Obsessional Inventory Child Version* (LOI-CV) [39]. This scale assesses the existence of obsessive-compulsive symptoms and the extent of interference associated with them. The LOI-CV was previously used in populations with EDs [40]. A high correlation was found between the adult and the childhood versions of the LOI [41]. For reasons of brevity, we related in our study only to LOI-interference. The internal consistency of the LOI-CV in the present study was 0.96.

The Hebrew translations of these scales have been validated in Israeli ED samples (EDE-Q [34]; EAT-26, BDI, STAI [42]; EDI-2 [43]; LOI [44]).

ADHD symptoms have been assessed using the *Adult ADHD Self-Report* (ASRS) [45] designed for adults, and the *ADHD Rating Scale-IV-Home Version* (ADHD-RS) [46] designed for adolescents. The ADHD-RS was originally constructed to be filled by parents. For the purpose of this study, in order to be filled by the participants, we changed the phrasing from the third person to the first-person wording. A self-rating version of the ADHD-RS has also been used elsewhere [47,48]. Both scales include 18-items relating to DSM diagnostic criteria of ADHD, and have been previously validated in Israeli samples [49,50]. The ASRS has also been previously used in patients with EDs [51]. The ASRS includes one outcome measure, the total score, whereas the ADHD-RS consists of three outcome measures: inattention, hyperactivity/impulsivity, and total score. The internal consistency of the ASRS and the ADHD-RS in the present study was 0.95 and 0.93, respectively, and of the inattention and hyperactivity/impulsivity scales of the ADHD-RS, 0.90 and 0.85 respectively.

**Neurocognitive battery.** *A Conjunctive Continuous Performance Test* was used to assess sustained attention. Participants were presented with a sequence of color drawings, and were instructed to respond as soon as a target (red square) appeared, and to withhold responses to all other stimuli. Standard deviation (SD) of reaction times (RT) is considered a representative index for evaluating sustained attention [24,52].

*The Conjunctive Visual Search task* has been used to assess selective attention. Participants are required to search for a target (blue square) appearing among an equal number of red squares and blue circles. RT and accuracy data are used in calculating the index reflecting the effect of attentional load on performance [24,52].

*A peripheral cueing paradigm with exogenous cues* was used to assess orienting of attention. Participants were instructed to respond to a target-stimulus (circle or triangle), appearing inside a cued (a light flashing briefly) or an un-cued rectangle, located to the right or left of a fixation point. The performance ratio of response time to invalid-cue trials (the target is not on the cued rectangle), vs. valid-cue trials serves as an index of the ability to orient to stimulus location [23,53].

*A Location****-****Direction Stroop-like task* [24] was used to assess executive attention. Participants were presented with an arrow pointing up or down, above or below, the fixation point. The task was composed of location subtask and direction subtask. Half of the trials within each subtask were congruent (on the location and direction of the arrow) and half were incongruent. RT and accuracy data in the different conditions and in the different stimuli combinations were used in calculating the index reflecting the effect of conflict on performance [24,52].

The formulas for the calculation of all neuropsychological measures are described in Table 1.

**Table 1:** Calculation of neuropsychological measures.

Attention Function	Operational measure
Sustained attention	*SD of RT*
Selective attention	[3*(8*RT_*8*_*/Acc*_*8*_+16*RT_*16*_*/Acc*_*16*_+31*RT_*32*_*/Acc*_*32*_)-(8+16+32)* (RT_*8*_*/Acc*_*8*_*+ RT*_*16*_*/Acc*_*16*_*+ RT*_*32*_*/Acc*_*32*_)]/[3*(8^*2*^*+16*^*2*^*+32*^*2*^*)-(8+16+32)*^*2*^*]*
Orienting of attention	Task score RT = [RT(Invalid)-RT(Valid)]/[(RT(invalid)+RT(valid))/2]] Cue Benefit RT = [RT(Neutral)-RT(Valid)]/[(RT(Neutral)+RT(Valid))/2]] Cue Cost RT = [RT(Invalid)-RT(Neutral)]/[(RT(Invalid)+RT(Neutral))/2]]
Executive attention	*[(RTDir*_*incong*_*/AccDir*_*incong*_*+RTLoc*_*incong*_*/AccLoc*_*incong*_*)-(RTDir*_*cong*_*/AccDir*_*cong*_*+RTLoc*_*cong*_*/AccLoc*_*cong*_*)] /[mean(RTDir*_*incong*_*/AccDir*_*incong*_*+RTLoc*_*incong*_*/AccLoc*_*incong*_, *RTDir*_*cong*_*/AccDir*_*cong*_*+RTLoc*_*cong*_*/AccLoc*_*cong*_*)]*

Note: RT = reaction time; Acc = accuracy; SD = standard deviation; numbers 8, 16, 32—denote display sizes in the selective attention task; Dir = direction subtask; Loc = location subtask; Cong = congruent; Incong = incongruent.

**Neurocognitive battery—Data analysis.** To eliminate trials with exceptionally long latencies in the selective-, orienting- and executive-attention tasks, we calculated mean RTs for correct responses for each condition, after excluding trials in which RT exceeded 4000 ms, and trials in which RT deviated more than 2 standard deviations from the participant's mean RT. In accordance with previous studies [52,53], we extracted for the selective-, orienting- and executive-attention tasks a single summarizing measure—inverse efficiency index [IEI]. As the IEI included a skewed RTs distribution, it was transformed to a natural log, reflecting the performance efficiency of the corresponding attention function. Similarly, a log transformation was applied to the standard deviation of RT in the sustained attention task, to reduce the impact of extreme values and bring the distribution of RT closer to a normal distribution.

### Procedure

Participants and parents, in the case of minors under the age of 18, signed a written informed consent, after being explained about the aims of the study. The study was approved by the Ethics Review Board of the Sheba Medical Center. Patients were interviewed independently by experienced psychiatrists and child and adolescent psychiatrists. Diagnoses were confirmed in clinical meetings of the two departments. Testing for all participants was administered individually by a single researcher. The sustained attention task was always the first to be administered, whereas the other tasks were counter-balanced across participants. The self-rating questionnaires were distributed randomly after the completion of the neuropsychological battery. Height and weight were measured during the morning hours, using standardized procedures [54].

The questionnaires and neuropsychological battery were administered two weeks before discharge. To be discharged, patients with AN-R were required to maintain BMI of at least 19 kg/m^2^ for at least two consecutive weeks. Patients with BN were required to have no B/P behaviors for at least two consecutive weeks as assessed using daily food monitoring sheets. AN-B/P patients were required to fulfill both criteria. Controls were similarly assessed.

### Statistical analysis

Data analysis was performed using IBM SPSS Statistics for Windows, version 23.0 (Armonk, NY: IBM Corp). Correlations between the different attentional variables and the other variables introduced were computed using Pearson Correlation Coefficients for continuous variables. Comparisons between patients with and without binge-eating and controls, as well as between the different DSM-5 [16] diagnostic ED subtypes, were computed for most variables using univariate analysis of variance (ANOVA). Between-group differences in countries of birth and use of medications were analyzed using chi-square analysis. Post-hoc comparisons according to Bonferroni were used to indicate the specific differences among the groups. Analyses of covariance (ANCOVAs) were computed to assess whether the between-group differences in the neuropsychological tasks and self-rating ADHD questionnaires would be maintained controlling for the influence of relevant demographic and psychometric variables. Effect size for the ANCOVA was calculated according to partial Eta-squared. We used the Bonferroni method to control for multiple comparisons by multiplying the p value by the number of independent tests introduced into the analyses, keeping the common cutoff value of p < .05. The 5 independent tests introduced into the analyses were the SD of RT of sustained attention, and the four outcomes of the two ADHD self-rating scales.

## Results

### Three-group analysis

#### Demographic and clinical background

The age of patients with non-binge EDs (M = 18.36, SD = 3.1) was significantly younger than that of patients with EDs involving binge-eating (M = 20.67, SD = 4.35) and controls (M = 19.95, SD = 3.38) (F_(2,165)_ = 5.92, p = .003). In addition, although the mean BMI of patients with non-binge EDs (M = 19.85, SD = 1.28) was significantly lower (F_(2,165)_ = 9.57, p = .0001) than that of patients with binge-EDs (M = 21.40, SD = 2.58) and controls (M = 21.17, SD = 2.11), the mean BMI in all three groups (as well as the individual BMIs of all participants) were within normal ranges. Last, no significant differences were found between patients with and without binge-eating in the use of medications; 76.5% of the patients with EDs involving binge-eating were treated with antidepressants, 41.2% were treated with antipsychotics, and 21.5% with mood stabilizers. The percentage of patients with no binge-eating that were treated with antidepressants, antipsychotics and mood stabilizers were 83.1%, 54.2%, 13.6% respectively. None of the patients was taking any psychostimulant medication at the time of the study.

#### Assessment of psychometric characteristics

Table 2 summarizes the differences between the two research groups and controls in the various psychometric measurements introduced. For two of the ED-related questionnaires (EDE, EDI-2), data existed for 80% of the sample. The rate of missing data was similar for all three groups. The severity of the symptoms on the EDE, EAT-26, EDI-2-IA, EDI-2-IR, BDI, STAI-T, and LOI was higher in the two research groups vs. the controls (p < .00001).

**Table 2:** Psychometric variables: Three-group analysis (ANOVA).

	Controls (n = 58)		Binge EDs (n = 51)		Non-binge EDs (n = 59)		F_(2,165)_	p	ES
	mean	SD	mean	SD	mean	SD			
**BDI**	4.00^b^	4.97	24.57^a^	13.78	27.73^a^	15.21	64.76	< .00001	0.44
**STAI- Trait**	31.52^b^	7.71	56.24^a^	10.15	57.00^a^	11.56	121.56	< .00001	0.60
**LOI-Interference**	11.17^b^	7.34	33.14^a^	20.91	32.56^a^	20.38	29.98	< .00001	0.27
**EAT-26**	5.68^b^	4.64	35.69^a^	17.79	37.47^a^	19.40	76.07	< .00001	0.48
							F_(2,131)_		
**EDE-Q-Total**	0.58^b^	0.56	3.57^a^	1.36	3.60^a^	1.67	85.40	< .00001	0.56
**EDE-Q-Restraint**	0.56^b^	0.87	2.72^a^	1.57	3.08^a^	1.93	37.86	< .00001	0.37
**EDE-Q-Eating Concerns**	0.18^b^	0.32	2.98^a^	1.41	2.67^a^	1.62	70.44	< .00001	0.52
**EDE-Q-Weight Concern**	0.88^b^	0.85	4.65^a^	1.41	4.64^a^	1.71	117.61	< .00001	0.64
**EDE-Q-Shape Concerns**	0.71^b^	0.71	4.29^a^	1.46	4.02^a^	1.90	88.71	< .00001	0.58
**EDI-2- Impulse Regulation**	17.40^b^	5.27	35.51^a^	8.74	32.76^a^	9.08	69.67	< .00001	0.52
**EDI-2- Interoceptive Awareness**	18.43^b^	4.77	37.15^a^	8.39	35.00^a^	9.47	78.51	< .00001	0.55

Note: ED–eating disorder; BDI—Beck Depression Inventory; STAI—State-Trait Anxiety Inventory; LOI—Leyton Obsessional Inventory; EAT-26—Eating Attitudes Test-26; EDI-2 Eating Disorders Inventory-2; EDE- Q—Eating Disorder Examination Questionnaire. Means with different superscripts indicate significant between-group differences in that row. Means with the same superscripts are not different from each other in that row. ES = effect size (η^2^).

#### Correlational analyses

Significant correlations were found between the SD of RT of sustained attention and ADHD-RS-inattention (r = 0.38, p < .0001), ADHD-RS-hyperactivity/impulsivity (r = 0.32, p < .0001), the combined measure of the ADHD-RS (r = 0.35, p < .0001), and the ASRS (r = 0.21, p < .01). According to Table 3, significant correlations were found also between the ADHD-RS, ASRS, and the SD of RT of sustained attention and the EDE-Q subscales, the EAT-26, the EDI-2-Impulse Regulation and EDI-2-Interroceptive Awareness, and the scales assessing depression, anxiety and obessionality (r = 0.21–0.71; p < .05-.000). Higher ED-related and comorbid psychiatric pathology was correlated with greater severity of ADHD-related disturbance. In addition, the SD of RT of sustained attention was correlated with the patients' age (r = -0.42, p < .0001). Last, BMI was not correlated with any of the attention measures.

**Table 3:** Correlations among attention measures and ED symptoms, depression, anxiety, obsessionality, age and BMI.

	ASRS	ADHD-RS-IA	ADHD-RS-HI	ADHD-RS–combined	SD of RT Sustained attention	IEI Orienting attention	IEI Selective attention	IEI Executive attention
**BDI**	0.60****	0.63****	0.51****	0.62****	0.31****	-0.01	0.25***	-0.05
**STAI- Trait**	0.71****	0.68****	0.57****	0.67****	0.25***	-0.01	0.25***	-0.11
**LOI-Interference**	0.51****	0.53****	0.49****	0.55****	0.31****	0.05	0.22***	-0.11
**EAT-26**	****0.47	0.53****	0.48****	****0.54	0.32****	0.00	0.20*	-0.06
**EDE-Q-Total**	0.57****	0.48****	0.39****	0.47****	0.29***	0.04	0.17*	-0.10
**EDE-Q-Restraint**	0.45****	0.56****	0.56****	0.59****	0.30**	0.12	0.24**	-0.04
**EDE-Q-Eating Concerns**	0.55****	0.62****	0.59****	0.62****	0.21*	0.04	0.20*	-0.13
**EDE-Q-Weight Concern**	0.54****	0.62****	0.60****	0.64****	0.31***	0.00	0.10	-0.09
**EDE-Q-Shape Concerns**	0.54****	0.64****	0.61****	0.66****	0.29***	0.03	0.11	-0.14
**EDI-2- Impulse Regulation**	0.60***	0.65***	0.62***	0.67***	0.34****	0.07	0.11	0.04
**EDI-2- Interoceptive Awareness**	0.56***	0.66***	0.60***	0.67***	0.26**	0.09	0.13	0.00
**Age (years)**	0.00	-.07	-0.13	-0.10	-0.42****	0.11	0.03	-0.20*
**BMI**	-0.10	-0.10	-0.12	-0.12	-0.50	-0.08	-0.13	0.00

Note

*p < .05

**p < .01

***p < .001

****p < .0001

ED–eating disorder; ADHD-RS: Attention Deficit Hyperactivity Disorder Rating Scale; IA-Inattention; HI: Hyperactivity/Impulsivity; ASRS: Adult ADHD Self-Report. IEI: Inverse Efficacy Index; BDI—Beck Depression Inventory; STAI—State-Trait Anxiety Inventory; LOI—Leyton Obsessional Inventory; EAT-26—Eating Attitudes Test-26; EDE- Eating Disorder Examination Questionnaire; EDI-2- Eating Disorders Inventory-2; BMI- body mass index.

#### Comprehensive assessment of attention

Table 4 summarizes the differences between the three groups in the various measurements of attention after controlling for age, EAT-26, BDI, STAI-T and LOI-interference. The post-hoc analysis of covariance (ANCOVA) indicated that after controlling for these variables, the differences between the groups were found only in the ADHD-RS dimensions. Thus, greater severity of ADHD-RS attention symptoms was found in the binge- eating vs. the non-binge eating and control groups (p corrected by Bonferroni = .0003) and the two clinical groups showed elevated hyperactivity/impulsivity symptoms compared to the control group (p corrected by Bonferroni = .0034). In the combined ADHD-RS scale, a significant difference was found between all groups, with controls scoring the lowest, the non-binge eating group in-between, and the binge-eating group scoring the highest (p corrected by Bonferroni = .0001).

**Table 4:** Assessment of attention functioning: Three-group analysis (ANCOVA).

	Controls (n = 57)		Binge EDs (n = 49)		Non-binge EDs (n = 59)		F_(2,157)_	p	p corrected by Bon-ferroni	ES
	mean	SD	mean	SD	mean	SD				
**ADHD-RS-IA**	2.88^a^	3.09	12.45^b^	5.33	10.41^a^	6.22	10.33	.000061	.00030	0.116
**ADHD-RS-HI**	3.04^a^	2.86	10.47^b^	5.28	9.46^b^	5.33	7.63	.00068	.0034	0.089
**ADHD-RS–combined**	5.91^a^	5.54	22.92^b^	9.78	19.86^c^	10.48	11.09	.000031	.00015	0.124
**ASRS**	30.49	11.17	48.88	13.71	43.07	15.49	4.23	.0163	.0815	0.052
**SD of RT sustained attention**	3.99	0.26	4.19	0.31	4.28	0.33	3.07	.0492	.246	0.038

Note: ED: eating disorder; ADHD-RS: Attention Deficit Hyperactivity Disorder Rating Scale; IA-Inattention; HI: Hyperactivity/Impulsivity; ASRS: Adult ADHD Self-Report. SD: standard deviation; RT: reaction time. Means with different superscripts indicate significant between-group differences in that row. Means with the same superscripts are not different from each other in that row. ES = effect size (partial η^2^).

In the neuropsychological assessment, we did not find significant between-group differences in the IEI of the selective-, orienting-, and executive attention tasks. In addition, the significant between-group difference in the SD of RT of sustained attention did not maintain its significance following Bonferroni correction (see Table 4). Last, no significant differences were found for the prevalence of ADHD diagnosis between patients with (16.6%) and without binge-eating (13.6%) (χ2 = 0.73, p = .392).

As noted earlier, the classification of the participants according to the presence/absence of binge-eating created heterogeneous populations in the context of the different ED subtypes included in each category. Therefore, the measures of attention functioning were reanalyzed according to the specific DSM-5 [16] ED subtypes. We did not include the DSM-5 [16] category of PD in this analysis because of the small number of patients with PD (n = 7). This ED-subtype classification yielded three clinical groups: AN-R (n = 34); AN-B/P (n = 36), and BN (n = 33). Eighteen patients in the AN-B/P sample were diagnosed with AN purging type and 18 with AN binge/purge type. As no differences were found between the two groups in any of the variables introduced, we related to these patients as one group of AN binge/purge type (AN-B/P).

### Four group analysis- analysis according to DSM-5 ED subtypes

#### Demographic, clinical and psychometric variables

Table 5 summarizes the between-group differences in the demographic, clinical and psychometric variables according to the DSM-5 group analysis. Patients with AN-R were significantly younger than BN and control participants, and had a significantly shorter duration of illness than patients with BN. Patients with AN-R had a longer duration of inpatient treatment than patients with AN-B/P and BN. The BMI of patients with AN of both types was significantly lower in comparison to the two other groups. In addition, patients with AN-B/P scored higher on the depression and obsessionality measures than patients with BN and AN-R. On the measures of ED-related pathology, patients with AN-B/P scored higher than patients with AN-R, except for EDE-Q-restraint. Last, on the EDE-Q-Total and two subscales of the EDE-Q, patients with BN fared worse than patients with AN-R (see Table 5).

**Table 5:** Demographic, clinical and psychometric variables: Four-group analysis (ANOVA).

	Controls (n = 58)		BN (n = 33)		AN-B/P (n = 36)		AN-R (n = 34)		F_(3,156)_	p	ES
	mean	SD	mean	SD	mean	SD	mean	SD			
**Age (years)**	19.95^a^	3.38	20.88^a^	4.03	19.44^a,b^	4.21	17.72^**b**^	2.63	4.99	< .01	0.08
**BMI**	21.17^a^	2.11	22.08^a^	2.58	19.74^**b**^	1.16	19.63^**b**^	1.04	14.55	< .0001	0.21
									F_(2,100)_		
**Duration of illness (years)**	-	-	5.22^**b**^	3.15	4.77^**ab**^	3.00	3.47^a^	2.60	3.45	< .05	0.06
**Duration of hospitalization (days)**	-	-	134.52^**b**^	86.55	151.75^b^	86.53	207.82^a^	105.06	4.67	< .05	0.08
**BDI**	4.00^a^	4.97	23.28^b^	11.56	32.11^c^	16.49	23.59^b^	14.17	49.59	< .00001	0.47
**STAI- Trait**	31.52^a^	7.71	55.73^b^	10.19	59.33^b^	11.12	54.88^b^	11.20	83.81	< .00001	0.60
**LOI-Interference**	11.17^a^	7.34	28.75^b^	18.42	39.81^c^	21.23	30.24^b^	20.84	24.10	< .00001	0.30
**EAT-26**	5.68^a^	4.64	35.92^bc^	16.66	43.06^c^	19.45	30.94^b^	18.36	57.62	< .00001	0.52
									F_(3,123)_		
**EDE-Q-Total**	0.58^a^	0.56	3.78^c^	1.31	3.94^c^	1.35	2.87^b^	1.81	65.28	< .00001	0.60
**EDE-Q-Restraint**	0.56^a^	0.87	3.12^b^	1.65	3.15^b^	1.75	2.35^b^	1.90	26.90	< .00001	0.38
**EDE-Q-Eating Concerns**	0.18^a^	0.32	3.05^c^	1.26	3.20^c^	1.51	2.01^b^	1.63	56.38	< .00001	0.57
**EDE-Q-Weight Concern**	0.88^a^	0.85	4.63^bc^	1.52	5.25^c^	0.92	3.91^b^	1.97	91.12	< .00001	0.68
**EDE-Q-Shape Concerns**	0.71^a^	0.71	4.33^c^	1.45	4.70^c^	1.26	3.23^b^	2.13	71.10	< .00001	0.62
**EDI-2- Impulse Regulation**	17.40^a^	5.27	33.39^bc^	7.52	36.76^c^	8.85	31.58^b^	10.26	48.57	< .00001	0.53
**EDI-2- Interoceptive Awareness**	18.43^a^	4.77	36.13^bc^	8.22	38.93^c^	8.48	32.29^b^	9.55	58.27	< .00001	0.58

Note: BN: bulimia nervosa; AN-anorexia nervosa; R-restricting type; B/P: binge/purge type; BDI—Beck Depression Inventory; STAI—State-Trait Anxiety Inventory; LOI—Leyton Obsessional Inventory; EAT-26—Eating Attitudes Test-26; EDI-2—Eating Disorders Inventory-3; EDE- Eating Disorder Examination Questionnaire. BMI- body mass index. Means with different superscripts indicate significant between-group differences in that row. Means with the same superscripts are not different from each other in that row. ES = effect size (η^2^).

#### Assessment of attention functioning

As shown in Table 6, after controlling for age, EAT-26, BDI, STAI-T and LOI-interference, significant between-group differences were found in all three ADHD-RS scales (p corrected by Bonferroni < .01-.0001). Patients with BN and AN-B/P reported greater severity on ADHD-RS-inattention, ADHD-RS-hyperactivity/ impulsivity and the combined ADHD-RS scale than control participants. Patients with BN also scored higher on ADHD-RS-inattention and the combined ADHD-RS scale compared to patients with AN-R.

**Table 6:** Assessment of attention functioning: Four-group analysis (ANCOVA).

	Controls (n = 57)		BN (n = 32)		AN-B/P (n = 35)		AN-R (n = 34)		F_(3,149)_	p	p corrected by Bonferroni	ES
	mean	SD	mean	SD	mean	SD	mean	SD				
**ADHD-RS-IA**	2.88^a^	3.09	12.06^b^	5.51	12.89^b,c^	6.67	9.29^a,c^	5.05	7.38	.000065	.00032	0.137
**ADHD-RS-HI**	3.04^a^	2.86	9.94^b^	4.68	10.51^b^	5.75	9.29^,a,b^	5.45	4.96	.0026	.013	0.091
**ADHD-RS–combined**	5.91^a,d^	5.54	22.00^b,c^	9.39	23.40^b,c,d^	11.42	18.59^a,c,d^	9.46	7.59	.000092	.00046	0.129
**ASRS**	30.49	11.17	46.68	12.91	50.12	17.10	40.55	12.37	2.90	.037	.185	0.056
**SD of RT Sustained attention**	3.99^a^	0.26	4.10^a^	0.27	4.37^b^	0.35	4.22^a^	0.26	6.41	.00041	.002	0.114

Note: BN: bulimia nervosa; AN-anorexia nervosa; R-restricting type; B/P: binge/purge type; ADHD-RS: Attention Deficit Hyperactivity Disorder Rating Scale; IA-Inattention; HI: Hyperactivity/Impulsivity; ASRS: Adult ADHD Self-Report; SD: standard deviation; RT: reaction time.

Means with different superscripts indicate significant between-group differences in that row. Means with the same superscripts are not different from each other in that row. ES = effect size (partial η^2^).

No significant between-group differences were found on the IEI of the selective-, orienting-, and executive attention tasks. By contrast, the use of ANCOVA showed a between-group difference in the SD of RT score of sustained attention (p corrected by Bonferroni = .002). Specifically, the AN-B/P group scored significantly higher on this measure (indicating less efficient functioning) than the two other clinical groups and the controls (see Table 6). Last, although the between-group difference in ADHD diagnosis was not significant, a greater percentage of patients with AN-B/P had ADHD (28%) in comparison to patients with BN (12%) and AN-R (9%) (χ^2^ = 5.27, p = .072).

## Discussion

The aim of the present study was to investigate whether attention deficits are associated with binge-eating in patients with EDs. In line with our first hypothesis, we found that in comparison to controls, patients with and without binge-eating reported a higher frequency of ADHD-RS-hyperactivity/impulsivity and combined ADHD-RS symptoms, as well as of ED and comorbidity-related symptoms. However, in contrast to our second hypothesis, although patients with binge-type EDs reported more difficulties on the ADHD-RS-inattention and combined ADHD-RS scales than patients with non-binge EDs and controls, we did not find significant between-group differences in the neuropsychological attention assessment.

The inconsistency of the results obtained using the different evaluative attention instruments may suggest that they assess different attention-related domains. Thus, the information derived from self-report questionnaires reflects the participants' subjective evaluation of their attentive and organized everyday behaviors. By contrast, the efficacy measures of the four attentional functions used in this study are objective—based on mathematical formulas that take into consideration response times and accuracy rates in conditions posing different attention demands [23].

The finding of more difficulties in patients with EDs on the self-reported ADHD-RS compared with controls and with nonclinical norms [51], despite the lack of impairment in neuropsychological attention tests, is of interest. Self-reported attention difficulties may be found also in patients with depressive and anxiety disorders [55,56], both being comorbid with EDs [57]. The significant positive correlations found in our study between the results of the questionnaires evaluating behavioral symptoms of ADHD and those evaluating behavioral symptoms of EDs, depression, anxiety, and obsessionality (see Table 3) support this line of reasoning.

In contrast to previous studies [58], we suggest that self-reported attention difficulties in patients with EDs do not necessarily represent genuine neurocognitive impairments in basic attention functions, but may be rather associated with elevated ED-related and comorbid psychiatric impairment. Moreover, the pattern of correlations reported in our study indicates only relatively weak relations (.34>r>.20) between factors hypothesized to contribute to binge-eating behaviors (restricting eating, impulsiveness, depression, and anxiety) [59,60,61,62] and objective attention functions, as evaluated with the neuropsychological tests. By contrast, medium to large positive correlations were found between the subjective reports of attention difficulties and the severity of the ED-related and comorbid variables (.71>r>.47). In other words, an increase in the severity of restricting eating, impulsivity, depression, and anxiety has been found to be significantly correlated with an increase in the subjective report of difficulties in attending to daily tasks, despite the relatively weak association found between these factors and basic attentional functioning as measured using neuropsychological tests.

Patients with binge-eating have reported greater severity of attention difficulties on the ADHD-RS questionnaire than patients with no binge-eating. Nonetheless, the two groups include patients with different ED types. Most non-binge-eating patients (n = 34) have AN-R, whereas most of the patients with binge-eating (n = 33) have BN. In contrast to patients with AN-R, those with BN are characterized with impulsivity, novelty/sensation seeking, heightened reactiveness to stress, and emotional dysregulation [57,63,64]. These characteristics may explain why patients with BN may encounter more difficulties than patients with AN-R in everyday tasks requiring attention. Furthermore, the frequent oscillation in patients with BN between binge/purge and restricting eating behaviors may lead to an overall disorganized lifestyle and limit the individual’s ability to carry out routine everyday tasks requiring sustained attention [65].

Most importantly, we found that in the four-group analysis (according to the DSM-5 [16] ED criteria), patients with AN-B/P were different from all other groups in showing impaired performance on the sustained-attention task. This finding on sustained attention vs. the other attention tests is probably associated with it being the most frequently disturbed attention function in patients with ADHD [23]. The contention of actual disturbance in sustained attention in AN-B/P vs. other ED types is promising. Nonetheless, similar to previous research [57,64], these patients showed also a greater disturbance than patients with AN-R and BN in comorbid depressive and obsessional parameters (see Table 5). This may suggest that the impaired functioning of patients with AN-B/P on the sustained-attention task may be part of an overall more complex and severe clinical presentation.

Moreover, patients with AN-B/P have shown greater disturbance in the restrictive components of the EDE-Q (higher EDE-Q total score) and on the EAT-26 than patients with AN-R. In addition, although the BMI of all ED groups assessed in the stabilization phase is within normal limits, it is still lower in patients with AN-B/P vs. BN (see Table 5). In other words, although malnourishment is known to occur also in BN [66], it is likely more severe in patients with AN-B/P.

Altogether, these findings may suggest that the potentially more severe malnourishment and ED-related disturbance in patients with AN-B/P may be associated with the poor sustained attention in this group. In other words, poor sustained attention in AN-B/P might represent a state-dependent condition associated with an overall more severe ED-related pathology.

Nevertheless, our patients were assessed when achieving their required weight, and when not having pathological eating-related behaviors for at least two consecutive weeks. The occurrence of disturbed sustained attention in these stabilized conditions only in patients with AN-B/P may point to the possibility of it being a core neurocognitive trait specific of this subgroup, above and beyond the influence of malnourishment [67]. The finding that patients with AN-B/P show a trend of having a greater percentage of ADHD diagnosis in comparison to BN and AN-R patients, although this between-group difference is not significant, may support this line of reasoning. Nonetheless, longitudinal studies have to be carried out to shed more light on this uncertainty.

Reviewing the literature, we found one recent study assessing sustained attention in AN [68]. This study did not find a difference in sustained attention between patients with AN and controls. However, there was no mention as to subtyping the patients in this study to restrictive vs. binge/purge pathology. It is of note, in this respect, that a previous study of our group [69], found a significant difference in attention bias patterns between patients with AN-R and AN-B/P, an issue not investigated in previous studies assessing attention bias in AN.

The limitations of the current study include the use of a cross-sectional design that prevents the examination of causality. Second, the non-binge ED group is heterogeneous, including patients with AN-R and patients with purging behaviors. To overcome this limitation, we have reanalyzed our findings using a categorization of the patients by specific ED subtypes. Another limitation pertains to the severity of the illness in inpatients with EDs at the time of discharge from inpatient treatment. Thus, the findings would have been likely more robust if we had studied the patients in the acute phase of their illness. Nonetheless, we have been particularly interested to find out whether "trait-related" attentional deficits would be associated with the susceptibility to binge-eating, when actual binge-eating behaviors are supposed not to be present. In addition, in the acute phase of the disorder there is also greater severity of comorbid symptoms, such as depression, anxiety, or obsessionality, that may have an influence on the patients' performance in the neurocognitive tests. Fourth, although the age range of the participants is only 13 years (15–28 years), our sample includes both adolescents and young adults. Nonetheless, it is conceptualized that brain maturity in children with ADHD, for example achieving the expected brain volume, usually occurs before the age of 12 [70]. Additionally, the age variable has been controlled in the ANCOVA analysis. Last, as we have examined only inpatients, our findings cannot be generalized to less severe ED populations.

In addition, as we have aimed to standardize the procedure, hence not to use different scales for adolescents and adults, we have decided to use two scales assessing ADHD, the ASRS for adults, and the ADHD-RS for adolescents, in both populations. Both scales relate to the 18 DSM-5 [16] items diagnosing ADHD, and show the same directionality in differentiating between patients with EDs and controls. In addition, both have been similarly correlated with the variables assessing the severity of ED and comorbid pathology. Nonetheless, following Bonferroni correction, only the ADHD-RS, but no the ASRS, has significantly distinguished between patients with binge/purge EDs and non-binge/purge EDs from controls (see Table 4), and among the different DSM-5 ED types and controls (see Table 6). While this finding is unexpected, it might, perhaps, reflect a different phrasing of the two scales. Thus, the ADHD-RS has been phrased by us as a sentence in the first-person mode ("I tend to…), whereas the ASRS is phrased as a question in the second-person mode ("Do you tend to…?"). Perhaps, answering in s first-person mode allows for a more definite, affirmative perception of the issue in question, enabling more robust findings, hence the possibility of greater between-group differences. These suggestions are only speculative and call for structured assessments of both rating scales in adolescent and adult populations with ADHD, to better assess possible differences between them.

To summarize, our results emphasize the importance of increasing the awareness of clinicians to symptoms common both to binge/purge type EDs and ADHD, specifically calling for the evaluation of ADHD in AN-B/P. Future studies should examine attentional functions in large ambulatory samples, from the acute stage of the illness to recovery, to better understand the role of attention deficits in the course and outcome of AN-B/P.
